# Ultrafast Mid-IR Laser Scalpel: Protein Signals of the Fundamental Limits to Minimally Invasive Surgery

**DOI:** 10.1371/journal.pone.0013053

**Published:** 2010-09-28

**Authors:** Saeid Amini-Nik, Darren Kraemer, Michael L. Cowan, Keith Gunaratne, Puviindran Nadesan, Benjamin A. Alman, R. J. Dwayne Miller

**Affiliations:** 1 Program in Developmental and Stem Cell Biology, Hospital for Sick Children, University of Toronto, Toronto, Ontario, Canada; 2 Departments of Chemistry and Physics and the Institute for Optical Sciences, University of Toronto, Toronto, Ontario, Canada; Texas A&M University, United States of America

## Abstract

Lasers have in principle the capability to cut at the level of a single cell, the fundamental limit to minimally invasive procedures and restructuring biological tissues. To date, this limit has not been achieved due to collateral damage on the macroscale that arises from thermal and shock wave induced collateral damage of surrounding tissue. Here, we report on a novel concept using a specifically designed Picosecond IR Laser (PIRL) that selectively energizes water molecules in the tissue to drive ablation or cutting process faster than thermal exchange of energy and shock wave propagation, without plasma formation or ionizing radiation effects. The targeted laser process imparts the least amount of energy in the remaining tissue without any of the deleterious photochemical or photothermal effects that accompanies other laser wavelengths and pulse parameters. Full thickness incisional and excisional wounds were generated in CD1 mice using the Picosecond IR Laser, a conventional surgical laser (DELight Er:YAG) or mechanical surgical tools. Transmission and scanning electron microscopy showed that the PIRL laser produced minimal tissue ablation with less damage of surrounding tissues than wounds formed using the other modalities. The width of scars formed by wounds made by the PIRL laser were half that of the scars produced using either a conventional surgical laser or a scalpel. Aniline blue staining showed higher levels of collagen in the early stage of the wounds produced using the PIRL laser, suggesting that these wounds mature faster. There were more viable cells extracted from skin using the PIRL laser, suggesting less cellular damage. β-catenin and TGF-β signalling, which are activated during the proliferative phase of wound healing, and whose level of activation correlates with the size of wounds was lower in wounds generated by the PIRL system. Wounds created with the PIRL systsem also showed a lower rate of cell proliferation. Direct comparison of wound healing responses to a conventional surgical laser, and standard mechanical instruments shows far less damage and near absence of scar formation by using PIRL laser. This new laser source appears to have achieved the long held promise of lasers in minimally invasive surgery.

## Introduction

Lasers are capable of cutting with a spatial resolution at the fundamental limit for surgery –the single cell. However, the process involves ablation with resulting thermal and shock wave induced damage that extends well beyond the ablation zone. We recently reported on a new mechanism for laser induced ablation using strongly absorbed infrared pulses specifically tuned to IR active vibrations with pulse durations sufficiently short to drive ablation faster than thermal and acoustic transport induced damage, but long enough to avoid the ionizing radiation effects of plasma formation [1]. This optimized energy deposition process should represent the most efficient mechanism possible for cutting biological tissue with minimally induced damage. We have verified this assertion through comparative wound healing studies based on the assessment of scar tissue formation and analysis of the TGF-beta and ß-Catenin signalling pathways connected to the extent of scar tissue formation, using a conventional laser and mechanical surgical tools as references.

Skin wound healing is a regenerative process requiring the coordinated regulation of a variety of cell types and cell signalling pathways [2]. This healing process is comprised of overlapping and linked phases: inflammation, proliferation (new tissue formation), and tissue remodelling [3]. Coordination of these phases, together with cellular responses to tissue damage, shapes the outcome of healing tissue, resulting in a scar [3]. During the proliferative phase of wound healing, mesenchymal (fibroblast-like) cells migrate into the healing wound, proliferate, and produce a disorganized matrix, providing the initial tensile strength to the wound, and regulating the size of the scar that will form [4].

TGF-ß [5] and ß-Catenin [6] signalling pathways have been identified as major regulators of the proliferative phase of wound healing and consequently scar size [7], [8]. While most wounds heal with a scar that is acceptable to the patient, large scars cause considerable functional and cosmetic deformities, as well as psychological stress, and patient dissatisfaction. The biggest problem is the formation of scar tissue that impairs function, a problem in nearly all surgeries to some extent. Currently available approaches to optimize wound repair include refinements in surgical technique, nutritional supplementation, and the use of local wound care modalities [9]. Despite these approaches, there has been little progress in the ability to regulate wound size.

The laser was first used as a surgical tool shortly after its invention as an alternative to mechanical surgical tools [10]. In principle, lasers offer the prospect of performing surgery at the fundamental limit by exploiting the spatial phase coherence of laser radiation to focus sufficient intensity for ablation or cutting at the single cell level. Although lasers have emerged as a valuable surgical tool, conventional surgical lasers, having pulse durations longer than nanoseconds, impair the proliferative phase of healing due to thermally-induced cell damage in the surrounding tissue [11]. Conventional medical lasers show benefits over mechanical surgical tools only in a very limited number of procedures [12].

We recently reported on a novel laser source, the Picosecond IR Laser (PIRL), explicitly designed to exploit a newly discovered ablation mechanism in which the selective excitation of water's vibrational modes couples directly to translation motions within tissue, the very motions involved in ablation, faster than any other material. By achieving superheating on picoseconds timescales, the nucleation sites for the ablative phase transition have nanometer (molecular) dimensions, avoiding cavitation and associated shock wave induced damage that has been one of the major stumbling blocks in using lasers for surgery. The strong acoustic attenuation at the 100 GHz frequency range further ensures that all the absorbed photon energy ablates tissue on time scales much faster than heat transfer can damage adjacent tissue of the targeted area. The pulse duration and heating rate is also specifically designed to avoid multiphoton ionization effects [13], that lead to highly reactive species known to be a major problem with other ionizing radiation sources. Using the PIRL system as a surgical tool and comparing it with a conventional laser and mechanical surgical tools, we performed a wound healing study on mice and compared the resultant ablative and tissue damaging characteristics, as well as the final impact on scar size.

## Results and Discussion

**Figure 1:**
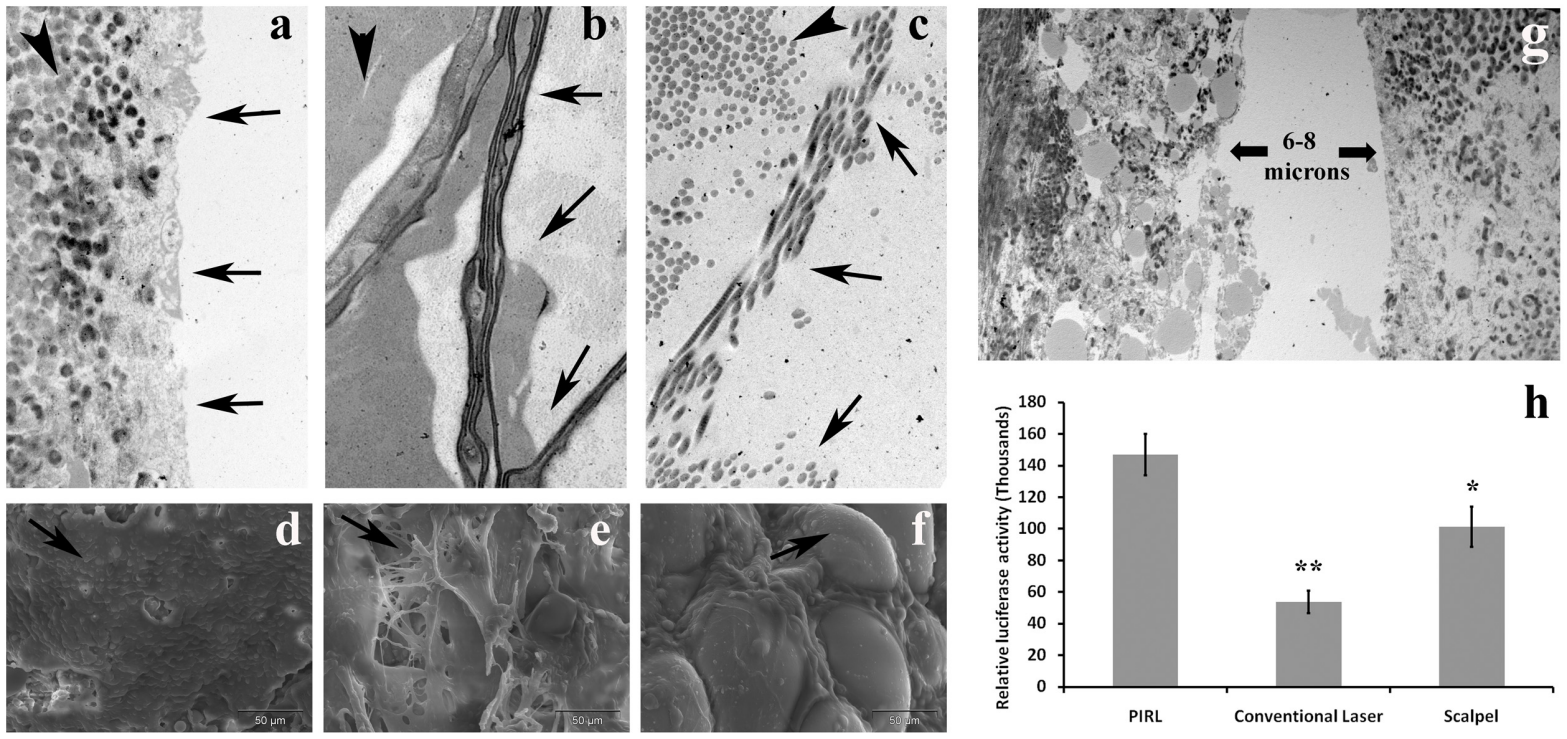
Minimal tissue ablation with less damage of surrounding tissues by using the PIRL laser. Transmission electron microscope (TEM) images of the incised skin. (**A**) PIRL laser (**B**) conventional laser (**C**) scalpel or the skin biopsy punch. Arrows show the incised edge of skin and arrowheads show adjacent tissues, which in PIRL laser incisions are intact. Scanning electron microscopy (SEM) of skin at the cut borders. (**D**) The PIRL laser kept the collagen layer intact. (**E**) The conventional laser damaged (burned) skin and deformed the collagen fibres resulting in a damaged, irregular extracellular matrix surface. (**F**) The scalpel damaged the skin by shearing between the collagen fibres and exposing individual cells (Arrow shows an adiopocytes which is exposed in this image). (**G**) The PIRL laser generated a cutting gap of 8 µm. (**H**) Comparison of relative number of viable cells extracted from same volume of skin. Harvested cells subjected to Luminescent Cell Viability Assay. Mean and 95% confidence interval of luciferase activity has been shown here. * P<0.01 and ** P<0.001 shows a significant differences compare to viable cells extracted from PIRL laser incisions.

To determine how the various modalities ablate tissue differently, the skin of the mouse subject was cut to a linear full thickness cut using the PIRL system, a commercial Er:YAG surgical laser (long pulse) at the exact same wavelength, or a conventional surgical scalpel. Transmission electron microscopy and scanning electron microscopy of the incised border revealed that the conventional laser damaged the skin border up to 800 µm away from the visible edge and the surgical scalpel caused dissociation of extracellular matrix fibres up to 400 µm further from the edge (Figure 1a–f). By comparison, cuts done with the PIRL system had sharp edges and minimal damage to adjacent tissue. The PIRL system generated a cutting gap of 8 µm, smaller than the diameter of a single skin fibroblast which was observed in the same skin sections. In contrast, the measured gap for scalpel incisions ranged from 40 to 120 micrometers and 650 µm for the conventional laser (Figure 1g). Wounds that were formed using the PIRL system had a higher number of viable skin cells immediately adjacent to the cut as compared to the other modalities (Figure 1h). Taken together, these results show that PIRL produces substantially less damage to the extracellular matrix and cells surrounding the wound, and ablates a much lower volume of tissue to execute the same function in comparison to a conventional laser and surgical scalpel.

**Figure 2:**
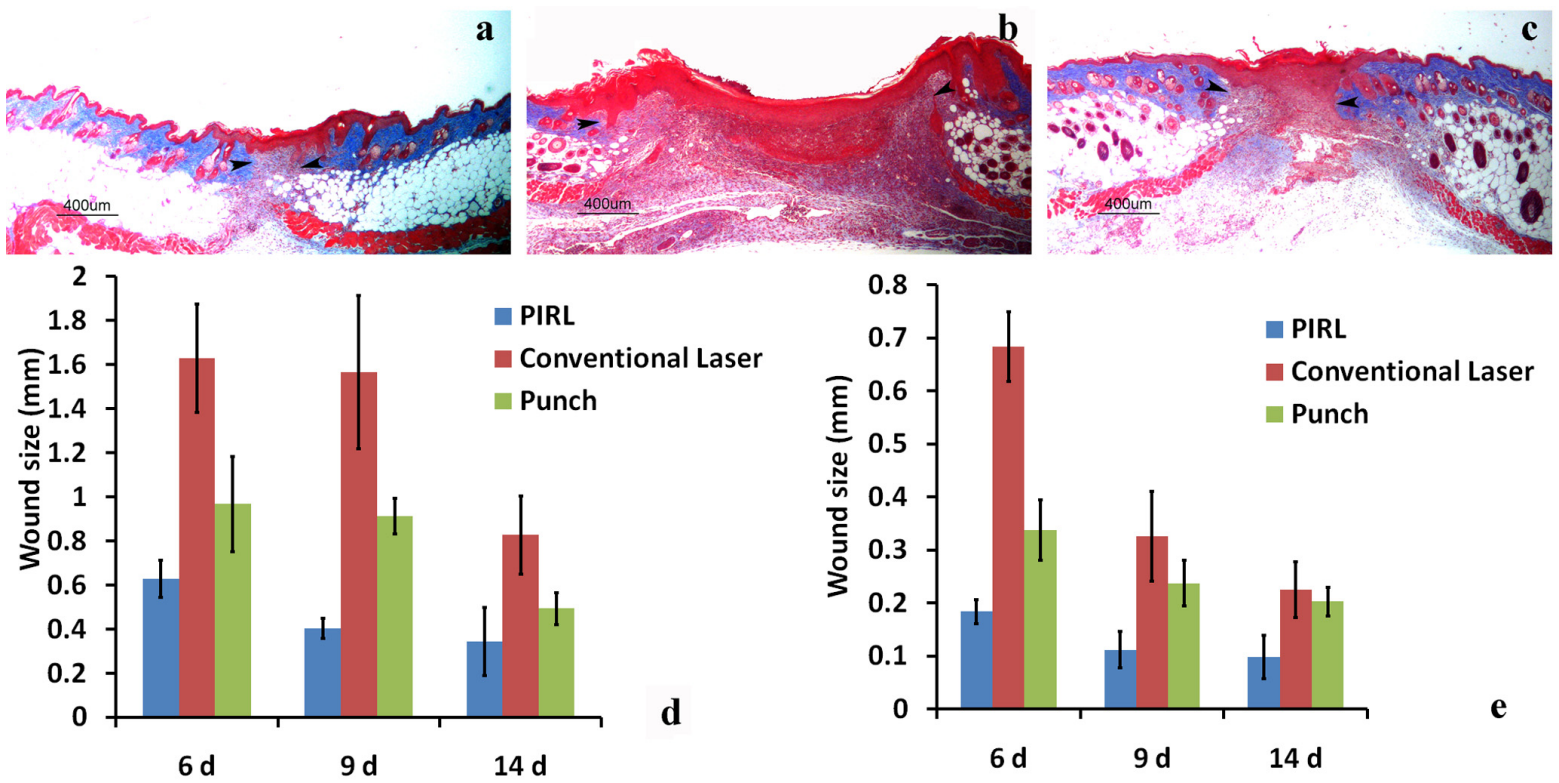
Minimal width of scars by using the PIRL laser. Representative histologic sections of healed skin of excisional 4 mm circular full thickness wounds using the three methods at 9 days post wounding. (**A**) PIRL Laser (**B**) Conventional Laser (**C**) Skin Biopsy Punch. Width of excisional (**D**) and incisional (**E**) wounds is given as the mean and 95% confidence interval of the maximal diameter of wounds. There was a significant differences in wound size between the three modalities, p<0.01. Arrowheads show the border of healing wounds with intact skins.

In order to evaluate the amount of tissue damage and its effect on scar formation, we removed the same amount of tissue (excision of 4 mm circular, full thickness, wounds) using the three methods and compared scar formation at different time points. Despite the same amount of tissue ablated by all modalities, the width of the scar formed by the PIRL system was half that of the wounds produced using either a conventional surgical laser or a scalpel at 9 days post-wounding (Figure 2a–d). A similar trend was observed when incision of linear wounds were performed (Figure 2e). Moreover, there was a lower proliferation rate, as measured using KI-67 staining and aniline blue staining showed higher levels of collagen in the early stages of wounds produced using the PIRL system, suggesting that these wounds mature faster, and thus have a shorter proliferative phase.

**Figure 3:**
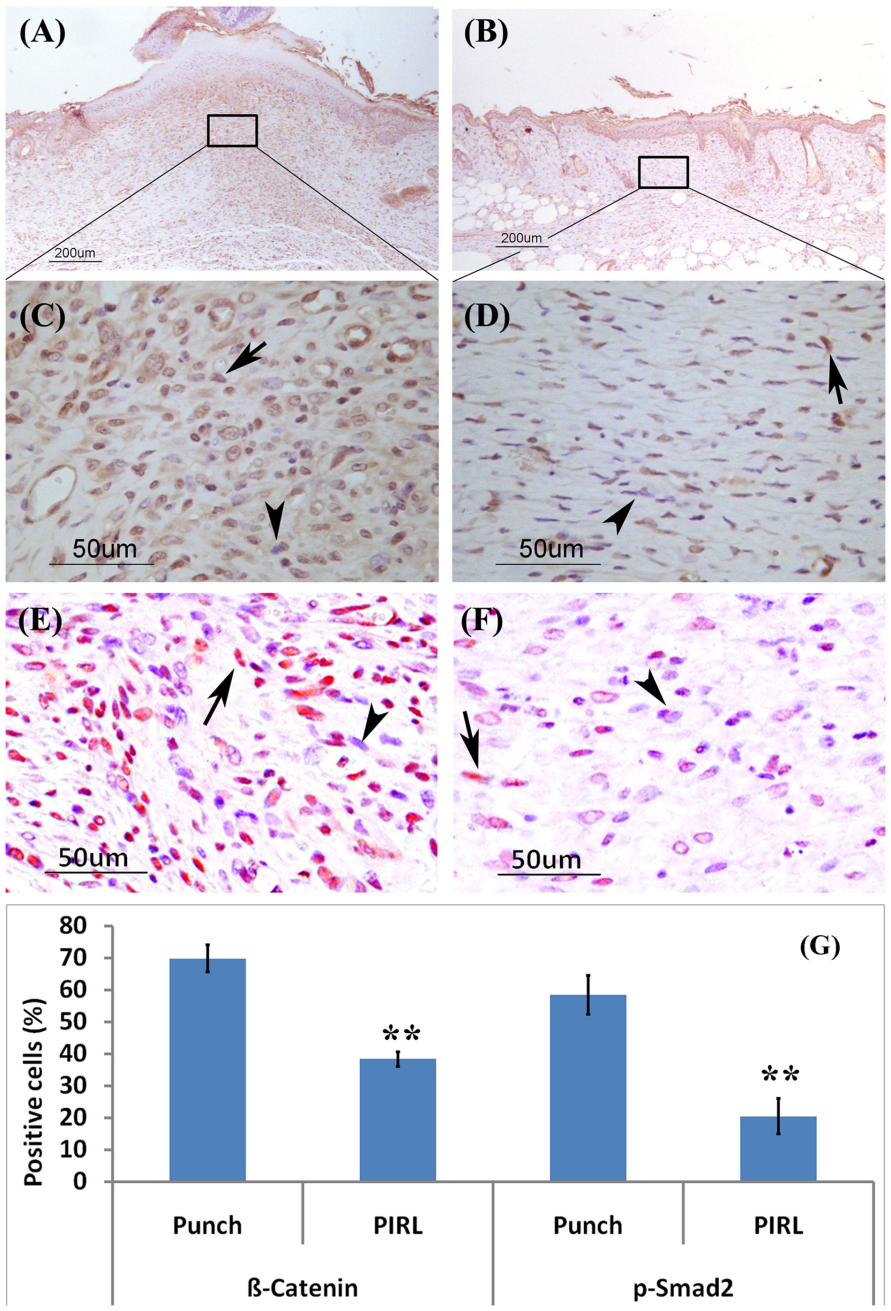
Less activation of β-Catenin and TGF- β signalling by using the PIRL laser. Immunohistochemistry for β-Catenin and phospho-Smad2 staining (9 days post wounding) using Rabbit Anti-Beta-Catenin antibody shows an increased number of stained cells for β-Catenin and phospho-Smad2 in punch wounds. (**A**) and (**C**) for β-Catenin, (**E**) for p-Smad2 in punch wounds. (**B**) and (**D**) for β-Catenin, (**F**) for p-Smad2 in the PIRL laser wounds. While 70% (+/−5%) of cells in punch wounds (**G**) are positive for β-Catenin, only 38% (+/−3%) in the PIRL laser created wounds stained for β-Catenin. The ratios for pSmad2 positive cells are 58%(+/−8%) and 20%(+/−7%) for mechanical and PIRL laser results, respectively. Mean and 95% confidence interval are given. Arrows show positive cells for either β-Catenin or p-Smad2 staining while arrowheads show negative stained cells.

Given the prominent role played by β-Catenin and TGF-β signalling in regulating wound size and tissue proliferation during wound healing, we compared the percentage of positive β-Catenin and pSmad2 cells between the three cutting methods. Here we found a significantly (P<0.001) lower number of positively stained cells in wounds produced using PIRL (Figure 3). This would suggest that differences in the surrounding tissue damage result in different cytokine profiles of the wounds.

**Figure 4:**
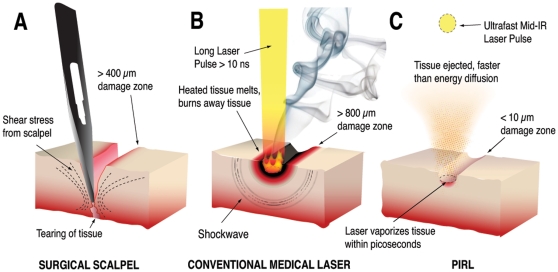
Schematic of cutting modalities. (**A**) The mechanical scalpel cuts skin by producing shear forces which exceed the elastic limit of the tissue. This causes a border of damage around the incision which reaches as far as 400 µm from the borders of the incision. (**B**) Conventional surgical lasers cut by depositing heat until the tissue melts or burns away. The damage zone in this case, can reach up to 800 µm away from the ablated edge. (**C**) By contrast, the well absorbed PIRL pulses cause superheating of water inside the tissue on the picosecond timescale, ejecting the tissue faster than energy can diffuse to the surroundings area. The remaining adjacent tissue shows minimal damage compared to the other two modalities.

These observations show that PIRL ablates the minimal amount of tissue and causes less damage to surrounding tissue (Figure 4), resulting in reduced activation of β-Catenin and TGF-β signalling, higher cell viability, lower cell proliferation and collagen deposition at an earlier time point in the scar, and thus an accelerated healing response. This selective ablation process owes its efficacy to the ultrafast time scale of the ablation process. The process occurs on timescales comparable or faster than even collision induced energy redistribution between molecules within the excited zone. We have observed whole proteins, even weakly bound protein complexes driven into the gas phase as intact neutral species using mass spectroscopy [14]. These molecules, especially the protein complexes, are extremely fragile and heretofore have never been observed in laser ablation without undergoing thermally driven fragmentation. This result shows that even at a molecular level there is minimal heat deposition into the constituent biological molecules. The key factor is the time scale under which the energy is preferentially partitioned within the excited water molecules that act as a propellant to drive the molecules into the gas phase and provide the cutting actions. The choice of pulse duration was made to be in this limit but not so short as to increase the peak power above the threshold for multiphoton ionization effects. In all cases, it is important to note that the forces remain far more localized than those involved in the use of mechanical tools, which need to exceed the shear elastic limit of the tissue in order to cut. During the proliferative phase of wound healing, cells undergo a transient phase of proliferation to fill the wound bed. Activation of β-Catenin and TGF-β signalling in this phase of repair are known to regulate wound size. The fact that PIRL does not significantly damage the extracellular matrix morphology, and the associated correlation with lower activation of the β-Catenin and TGF-β signalling pathways, indicates the different signalling levels arises from the minimized damage to the extracellular matrix. The effect in turn would limit the liberation of local cyctokines, and thereby expose the cells undergoing healing to different extracellular matrix signalling cues. These differences in the damage zone influence cell behaviour in a way that results in a smaller sized scar [15].

The use of PIRL can open up new surgical methods where scar tissue formation is particularly debilitating [16]. This approach may have general applications in reducing hyperplastic scarring and also cosmetic application in the revision of existing hyperplastic scars. Moreover by decreasing the healing time, this new surgical modality may result in increased patient comfort and decreased risk of infections due to infection in surgery. The PIRL system is a new tool for scar prevention, promising outstanding results and improved surgical outcomes. As stated by FitzGibbon [17]: “By your scars you will be judged”.

## Methods

### Ethics Statement

All procedures using animals were approved by the SickKids animal care committee (#7014, March 19^th^, 2009) under the auspices of Canadian Council on Animal care.

### Wound Healing

For excisional wounding, four full-thickness 4-mm diameter skin wounds were created on the dorsum of CD1 mice using the DELight Er:YAG laser system (emiting microsecond pulses at 2940 nm) and using computer controlled scanning optics for the PIRL system, with ∼300 ps pulses at 2950 nm or a dermal biopsy punch (Miltex Instrument Company, Bethpage, NY). Mice were euthanized at 3, 6, 9 and 14 days after wounding. For incisional wounding, six full-thickness 4 mm length linear skin wounds were created on the dorsum of CD1 mice using the two lasers and a conventional surgical scalpel. Mice were euthanized at 0, 3, 6, 9 and 14 days after wounding. Mice euthanized at time 0 were used for tissue ablation studies and the remaining tissue was used for scar formation studies. Eight animals were examined at each time point. Wound size was determined using histologic sections cut at a right angle to the skin surface across the wound. Serial sections were observed, and the section at the center of the excisional circular wound, with the largest wound diameter, was chosen to measure wound size. The edge of blue staining on mason trichrome (representing uninjured collagen) was taken as the wound edges in measuring the wound diameter. The maximal wound diameter was selected by measuring serial sections across the wound and taking the maximal width located at the center of the circular wound.

### Histochemistry and Immunohistochemsiry

Staining was performed on 5 µm formalin fixed paraffin embedded sections. Masson's Trichrome stain was performed to facilitate wound measurement. Following mordant in Bouin's solution for 20 minutes at 56°C, sections were stained in a stepwise manner with Weigert's Iron Hematoxylin, Biebrich Scarlett Acid Fuschin, phosphotungstic/phosphomolybdic acid solution, and aniline blue, followed by acetic acid. To assess the degree of collagen deposition in the tissue semi-quantitatively, a modified Masson's trichrome procedure was used. Following mordant in Bouin's solution for 20 minutes at 56°C, sections were stained with phosphotungstic/phosphomolybdic acid for 5 minutes, aniline blue dye for 8 minutes, and 1% acetic acid for 2 minutes. The degree of blue staining in the dermis layer was scored from 0–10 by two independent, blind observers. Several immunohistochemical stains were performed following antigen retrieval by pressure cooking for 4 ½ minutes in a 10.0 mM citrate buffer (pH 6). β-Catenin was detected by incubated with a primary β-Catenin antibody (Millipore Rabbit Anti β-Catenin polyclonal IgG) at 1∶200 dilution overnight at 4°C. Following incubation with the secondary antibody (Vector Labs Biotinylated Goat Anti-Rabbit IgG) at 1∶200 dilution for 30 minutes at room temperature, staining was developed with the chromogen 3,3′-diaminobenzidene from Vector Laboratories (Burlingame, CA, USA) for 2 minutes. Hematoxylin was used as a counterstain (45 seconds). Nuclear and cytoplasmic staining was quantitatively measured as the percentage of cells stained brown (in the nucleus, cytoplasm, or both) in a 20× field. Nuclear Phospho-Smad2 was detected by the same procedure, using Phospho-Smad2 primary antibody (Cell signalling: Phospho-Smad2 (Ser465/467) Rabbit mAb) and same secondary antibody used for β-Catenin, and scored similarly. KI-67 (a nuclear marker of proliferating cells) staining was performed following antigen retrieval by boiling for 20 minutes in a 1.0 mM EDTA buffer. Incubation and detection were performed as before, using Dako Monoclonal Rat Anti-Mouse KI-67 Antigen TEC-3 IgG2a primary at 1∶50 dilution for 1 hour at 37°C and Biolegend Biotin Goat Anti-Rat IgG Poly 4054 secondary at 1∶200 dilution for 30 minutes at room temperature. Nuclear staining was measured quantitatively as before.

### Statistical analysis

T-student analysis has been used to statistically analyse reported significances. To estimate the statistically significant sample size, we performed a power calculation to determine the number of animals that would be needed in each group to detect a 25% difference in wound size, a difference that is biologically and clinically significant. The mean wound size and the variability in wound size were taken from our previous studies examining wound size using the same wounding and analysis technique used previously in the same mouse strain [8]. We calculated that 8 mice would be needed in each group to detect this difference.

### Scanning electron microscopy

Scanning electron microscopy was used to examine the morphological characteristics of incised skin, using the three methods. CD1 mice were subjected to incision and the tissues immediately were fixed in 2% glutaraldehyde in 0.1 M sodium cacodylate (pH 7.3). After washing in buffer, tissues were dehydrated in a graded series of ethanol solutions. After critical point drying (Bal-Tec CPD 030), the samples were sputtered with gold (Denton Desk II sputter coater), and the probes were examined by scanning electron microscopy (FEI XL30 ESEM).

### Transmission electron microscopy

Freshly incised skin was fixed in 2% glutaraldehyde in 0.1 M sodium cacodylate buffer (pH 7.3). After being washed in buffer, the tissue samples tissues were post fixed in 1% osmium tetroxide in 0.1 M sodium cacodylate buffer, washed again then dehydrated in a graded series of ethanol solutions followed by infiltration and embedding in epon. Sections of 70 nm were cut and picked up on copper grids. The sections were stained with uranyl acetate and lead citrate and viewed with an FEI Tecnai 20 TEM. Images were recorded using an AMT 4000 digital camera.

### Viability Assay

One square centimetre of skin was subjected to 10 cross-sectional incisions using the three modalities. The remaining pieces of skins were digested using collagenase, dispase and trypsin for 30 minutes in a 37 C rotating incubator. After washing, cells were subjected to CellTiter-Glo® (Promega Corporation) substrate in order to measure the number of viable cells. Luminescence was measured with a Wallac Victor™ spectrophotometer after 10 minutes of incubation to determine relative cell viability.
